# Impact of perception reduction of employment opportunities on employment pressure of college students under COVID-19 epidemic–joint moderating effects of employment policy support and job-searching self-efficacy

**DOI:** 10.3389/fpsyg.2022.986070

**Published:** 2022-10-19

**Authors:** Shiyuan Yang, Jinxiu Yang, Longhua Yue, Jingfei Xu, Xingyu Liu, Wei Li, Hao Cheng, Guorong He

**Affiliations:** ^1^School of Humanities, Sichuan Agricultural University, Ya’an, China; ^2^School of Economics, Sichuan Agricultural University, Chengdu, China

**Keywords:** employment pressure, employment policy support, COVID-19 epidemic, perception reduction of employment opportunities, job-searching self-efficacy

## Abstract

Based on the stress interaction theory, this research constructed a model to study the joint moderating effects of the perception reduction of employment opportunities under the COVID-19 epidemic on the employment pressure of college students. With two moderating variables introduced, employment policy support and job-searching self-efficacy, this research studied the mechanism and boundary conditions of perception reduction of employment opportunities on employment pressure of college students from both individual and environmental aspects. The study found that during the epidemic if college students perceived fewer employment opportunities, they could have greater employment pressure from themselves, schools, and families; and that under the joint moderation of employment policy support and job-searching self-efficacy, the perception reduction of employment opportunities under the COVID-19 epidemic, the employment pressure of college students, universities, and families were connected, with different adjustment mechanisms. Based on empirical data, this research can provide theoretical enlightenment and practical guidance for the government, universities, and families to alleviate the employment pressure on college students during the epidemic.

## Introduction

Employment pressure has become the biggest psychological pressure on Chinese college students (Liu, 2019). Employment pressure will hurt college students’ physical, mental health, and subjective wellbeing (Liu, 2011; Deng et al., 2015; Jin et al., 2020). If the employment pressure is too high or it lasts for too long, it may also lead to serious psychological and mental diseases such as depression, insanity, neurosis, and a series of behavioral problems such as nightmares, insomnia, and even suicide (Tang and Sun, 2021). In 2021, the global working hours reduced by 4.3% compared to pre-epidemic levels, which is equivalent to a reduction of 125 million full-time jobs. This will directly cause the loss of about 700,000 new jobs in cities and towns and have a great impact on the employment of college students (Cheng, 2020). *A report on the Employment of College Students in the Fall of 2020* released by the Zhilian Research Institute points out that nearly 60% of students felt employment pressure and were willing to lower their salary expectations.

Previous research on employment stress of college students has mainly focused on the influencing factors of college students’ employment stress (Tang and Sun, 2021), the relationship with subjective wellbeing (Liu, 2019), physical and mental health (Chen, 2010), and coping strategies for employment stress (Guo et al., 2013; Yang et al., 2022) and other aspects, the impact of the epidemic on the employment pressure of college students is less explored. Stress is the product of a “force field,” an environment that all individuals and organizations confront with reinforcing or opposing forces (Smith, 1951). Under normal circumstances, the driving force and inhibitory force affecting the employment pressure of job seekers are balanced. However, the outbreak of the new crown epidemic has caused a strong impact on the labor market, the labor demand has weakened significantly, and the available jobs have also decreased. The increased inhibition will eventually break the balance of the “force field,” which will easily cause greater psychological pressure on job seekers. Li (2020) found that after the outbreak, both objectively and subjectively, the employment pressure on college students is on the rise. However, the current study does not explain the specific impact of the perceived reduction of employment opportunities under the COVID-19 epidemic on the employment pressure of college students. The question that this study seeks to explore.

According to the Stress Interaction Theory, stress is a special relationship between individuals and the environment that causes fatigue or exceeds individual psychological resources and endangers individual health (Lazarus, 1966; Lazarus and Folkman, 1984; Lazarus et al., 1985). College students’ employment pressure is a psychological tension phenomenon caused by the interaction of internal and external environment and personal factors in the employment situation (Liu, 2010). As a major social event, COVID-19 is an anticipatory stressor that may have a significant impact on college students. College students who are sensitive to changes in the labor market will have a stronger sense of urgency and feel greater employment pressure. From the perspective of the external environment, the employment of college students has always been the government’s focus on people’s livelihood issues. The State Council of China has issued the Implementation Opinions on Strengthening Employment Measures in Response to the Impact of the Epidemic, adopting various employment support policies to alleviate the impact of the epidemic on the employment of college students. Relevant employment support policies mainly include encouraging enterprises to expand the scale of recruitment, expanding the scope of grassroots employment, extending the length of employment internship, appropriately delaying the acceptance of employment, and providing employment subsidies to impoverished college students. Many universities have also taken active measures to support the employment of college students. Perceptions of reduced employment opportunities vary from person to person. People who are sensitive to changes in the labor market will have a stronger sense of urgency and feel greater employment pressure. Research shows that active employment support policies can help job-seekers improve matching efficiency to return to work quickly, and relieve employment pressure (OECD, 1993). At the same time, according to the Self-efficacy Theory, a high level of self-efficacy can help individuals generate positive beliefs and enable them to have the ability and courage to face pressure (Guo and Jiang, 2008). In the context of the labor market being hit by the COVID-19 epidemic, with highly effective employment support policies from the government, college students with high job-searching self-efficacy are more likely to find jobs efficiently, thereby weakening the impact of reduced employment opportunities on students’ employment pressure.

**FIGURE 1:**
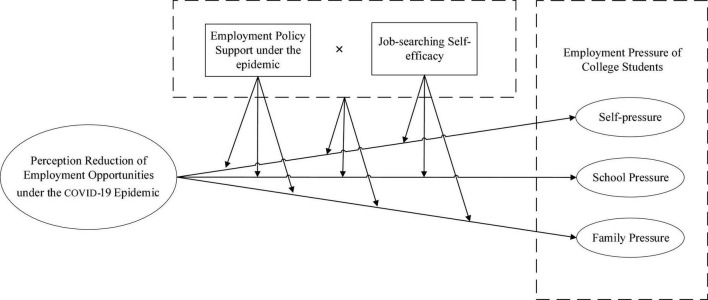
The mechanism model between college students’ perception reduction of employment opportunities and their employment pressure.

Based on this, this research studies the mechanism of the impact of college students’ perception reduction of employment opportunities under the COVID-19 epidemic on their employment pressure, and the contingency impact of employment policy support and job-searching self-efficacy. It is empirically tested with the survey data from 810 fresh college graduates employed in 2021. This study adopts the perception reduction of employment opportunities as an independent variable, employment pressure as a dependent variable, employment policy support and job-searching self-efficacy as moderating variables, and explores (1) the relationship between college student’s perception reduction of employment opportunities under the COVID-19 epidemic and their employment pressure; (2) employment policy support and job-searching self-efficacy and their combined effects on college students’ perception reduction of employment opportunities and employment pressure (Figure 1).

## Theory and hypotheses

### Related concepts

#### Perception reduction of employment opportunities under the COVID-19 epidemic

Employment opportunity refers to the possibility of an individual getting a job opportunity (Wang, 2018). Perception is an individual’s subjective feelings in the process of situational experience, and the individual’s subjective evaluation and judgment of things (Wang, 2016). Perception of employment opportunity refers to the degree of sensitivity of job seekers to changes in employment opportunities in the labor market (Gerhart, 1989), that is, a job seeker’s judgment on whether to obtain a job and the quality of the job through the evaluation of his employability and the experience of the employment environment. The perception reduction of employment opportunities under the COVID-19 epidemic refers to the feelings and judgments of job seekers about the degree of reduction in the target job opportunities under the background of the COVID-19 epidemic and a not optimistic employment environment. Different individuals have different feelings and judgments on whether they can get the target job and the quality of the job obtained through the evaluation of their employability and the experience of the employment environment. Faced with the same labor market conditions, the perception reduction of employment opportunities varies. This variable focuses on the subjective feelings of college students about employment opportunities in the labor market, and it will lead to different degrees of employment pressure of college students.

#### Employment pressure of college students

Stress is a special relationship between individuals and the environment that causes fatigue or exceeds individual psychological resources and endangers individual health (Lazarus et al., 1985). Employment pressure refers to the interaction process of an individual’s characteristics and personal inclination with the situational variables in the employment situation in the process of employment, which makes individuals feel nervous and anxious (Liu, 2010). College students’ employment pressure is a psychological tension phenomenon caused by the interaction of internal and external environment and personal factors in the employment situation (Liu, 2010). Lin et al. (2004) attribute the employment pressure of college students to eight aspects: the school reputation, the social environment, student’s academic performance, the desire of finding a good job, social relations, parents’ employment expectations, the major mismatch, and the social adaptability. Yan (2004) believes that the pressure of employment mainly stems from the social environment, the school education, family expectations, and graduates’ understanding of society and themselves. This study applies Liu’s (2010) method of dividing the sources of employment pressure of college students, that is, to attribute the employment pressure of college students to three aspects, namely family pressure, school pressure, and self-pressure.

#### Job-searching self-efficacy

Self-efficacy describes the phenomenological characteristics of a subject, which is a self-phenomenon and has the characteristics of self-evaluation. Its formation is based on people’s processing and weighing of various information reflecting their abilities, and it results from people’s perception of what they can do. Self-efficacy is people’s cognition and evaluation of their ability to perform specific tasks (Bandura, 1997). As a kind of self-evaluation and belief formed on this basis, self-efficacy plays a decisive role in the individual’s mental function and potential realization. Job-searching self-efficacy refers to the cognition and evaluation of a job-seeker’s ability to find a job (Trougakos et al., 2007).

#### Employment policy support under the COVID-19 epidemic

Employment policy refers to the government’s policies aimed at increasing labor demand and promoting employment. It is a package of measures usually adopted by the government to relieve employment pressure (OECD, 1964). Common active labor employment policies include direct job creation, public employment services or agents that help people search for jobs, training for unemployed people, employment subsidies for manufacturers that hire unemployed individuals, etc. Relevant policies for college students mainly include broadening students’ employment channels, encouraging enterprises to expand the scale of recruitment, expanding the scope of grassroots employment, extending the length of employment internship, appropriately delaying the acceptance of employment, and giving employment subsidies to poor college students. The employment policy support in this study refers to the degree of employment support from the government and schools perceived by college students during the epidemic situation.

#### Relationship between the perception reduction of employment opportunities under the COVID-19 epidemic and employment pressure of college students

Wheeler et al. (2005) believe that employment opportunity perception is the number of available alternative job opportunities perceived by individual workers. In a labor market with the same degree of tightness, different job seekers have different perceptions of job opportunities. Generally, job-seekers who are more sensitive to the reduction of job opportunities in the labor market will bear greater employment pressure; conversely, the less sensitive the perception reduction of employment opportunities, the less employment pressure they will bear. Hao (2020) believes that college students face employment pressure from families, schools, and themselves. Based on the Chinese University Student Tracking Survey (PSCUS), Li (2020) compares the changes in employment pressure, psychological pressure, and employment choices of fresh graduates before and after the outbreak of COVID-19. It is found that the COVID-19 epidemic situation has caused many negative impacts on the employment of graduates, such as blocked recruitment interviews, decreased job implementation rate, increased employment pressure, and pessimistic economic expectations for the future. Due to the impact of the epidemic, the communication and release of employment information between enterprises and universities have become untimely, and employment-related guidance that is supposed to carry out has been paused, making it more difficult for college students to grasp job information. In addition, many family members of college students are unable to resume work and production during the time of the COVID-19 epidemic, and their financial situation has become strained. This undoubtedly puts additional pressure on college students to be employed. Finally, the employment pressure on college students comes from the students themselves. Going to college is essentially an investment of human capital. The most basic goal is to obtain a return on this investment, and the only way of realization is whether a student can successfully graduate from college and get an ideal job. Therefore, the perception reduction of employment opportunities will undoubtedly affect all the above pressures. Accordingly, the following hypotheses are proposed:

H1: The perception reduction of employment opportunities under the COVID-19 epidemic has a positive impact on the employment pressure of college students. The higher perception reduction of employment opportunities perceived by college students, the greater their employment pressure will be.

H1A: The perception reduction of employment opportunities under the COVID-19 epidemic has a positive impact on the self-pressure perceived by college students.

H1B: The perception reduction of employment opportunities under the COVID-19 epidemic has a positive impact on the school pressure perceived by college students.

H1C: The perception reduction of employment opportunities under the COVID-19 epidemic has a positive impact on the family pressure perceived by college students.

### Contingency effect of the policy environment and job applicants’ characteristics

#### Contingency effect of employment policy support under the COVID-19 epidemic

Active Labor Market Policy, a labor market policy intervention, is aimed at increasing labor demand and promoting employment. Jorgen Elmeskov et al. (1998) investigated, recorded, and discussed the experience of active labor market policies in OECD countries, and pointed out that most countries have managed to adopt active labor market policies to solve the employment problem. These policies have achieved good results in some countries, such as Belgium, France, Germany, and other countries. After the outbreak of the COVID-19 epidemic, the Chinese government department has successively introduced many policies to promote the employment of college students, such as expanding the job supply of state-owned enterprises and public institutions, granting recruitment subsidies to small and medium enterprises (SMEs), targeted assistance to college graduates in areas severely affected by the epidemic, and launching large-scale “cloud recruitment” (i.e., online recruitment) *via* mainstream media. Research by Martin and Grubb (2001) shows that active labor market policies can increase the employment rate, bring hope to job seekers, and improve the welfare of the entire society. Fougère et al. (1998) adopted a job-searching model to study the effect of public employment agencies in France from 1986 to 1988. The study found that the improvement of the job-searching environment would reduce an individual’s effort to search for jobs and thus prolong the time of job searching. Obviously, with the same degree of reduction of employment opportunities, if an active labor market policy is implemented, an individual labor force will experience less employment pressure and vice versa. With an active labor market policy, job seekers with a high perception reduction of employment opportunities will also ease the employment pressure. In a labor market environment without active employment support policies, job seekers will be more anxious and stressed. Based on the above analysis, the following hypotheses are proposed.

H2: Under the epidemic, the employment policy support negatively moderates the relationship between the perception reduction of employment opportunities under the COVID-19 epidemic and employment pressure of college students. The greater the degree of employment policy support perceived by college students, the weaker the impact of their perception reduction of employment opportunities on employment pressure.

H2A: Under the epidemic, the employment policy support negatively moderates the relationship between the perception reduction of employment opportunities under the COVID-19 epidemic and the self-pressure of college students. The greater the degree of employment policy support perceived by college students, the weaker the impact of their perception reduction of employment opportunities on self-pressure.

H2B: Under the epidemic, the employment policy support negatively moderates the relationship between the perception reduction of employment opportunities under the COVID-19 epidemic and the school pressure of college students. The greater the degree of employment policy support perceived by college students, the weaker the impact of their perception reduction of employment opportunities on school pressure.

H2C: Under the epidemic, the employment policy support negatively moderates the relationship between the perception reduction of employment opportunities under the COVID-19 epidemic and the family pressure of college students. The greater the employment policy support perceived by college students, the weaker the impact of their perception reduction of employment opportunities on family pressure.

#### Contingency effect of job-searching self-efficacy

The employment pressure of college students is not only caused by the sense of incompetence at the threat of the employment environment but also caused by the ineffectiveness of self-control or the temporary disorder of psychological function. According to Bandura’s Self-efficacy Theory (Bandura, 1997), individuals with high job-searching self-efficacy seldom feel scared and helpless in the face of a labor market environment with reduced employment opportunities. A high level of job-searching self-efficacy can help job-seekers generate positive beliefs to face difficulties and alleviate pressure. Even if job seekers with high job-searching self-efficacy continue to suffer from job failures, their mentality can remain positive and their professional skills will not change much. They will always be willing to accept challenges, become more courageous as things turn tough, and not feel threatened. On the contrary, people with low job-searching self-efficacy will be vulnerable when faced with fewer opportunities in the labor market, and the experience of frustration in job hunting will have a greater impact on them. The research of Benati (2001) also shows that job-seekers constant employment failures will have a frustrating effect, and they may even withdraw from the labor market permanently. Other experiments (Schwarzer et al., 1992) also show that individuals with high self-efficacy, can bravely face failures and gain experience from them. Maybe it is because they have strong confidence in their professional skills, so they do not care about problems and changes in the outside world. They do not perceive more challenges and thus have less pressure. Individuals with low self-efficacy are more sensitive to changes in tasks and often do not have enough confidence to adapt and face the external environment. Due to their lack of self-confidence, they are more likely to be dominated by situational cues. Under the impact of the COVID-19 epidemic, the employment opportunities for college students have decreased, and the stronger the job-searching self-efficacy, the weaker the impact of the perception reduction of employment opportunities on the employment pressure of job-seekers, and vice versa. Based on the above analysis, this article proposes the following hypotheses.

H3: Job-searching self-efficacy negatively moderates the relationship between the perception reduction of employment opportunities under the COVID-19 epidemic and employment pressure of college students. The stronger the job-searching self-efficacy, the weaker the impact of their perception reduction of employment opportunities on employment pressure.

H3A: Job-searching self-efficacy negatively moderates the relationship between the perception reduction of employment opportunities under the COVID-19 epidemic and the self-pressure of college students. The stronger the job-searching self-efficacy, the weaker the impact of their perception reduction of employment opportunities on self-pressure.

H3B: Job-searching self-efficacy negatively moderates the relationship between the perception reduction of employment opportunities under the COVID-19 epidemic and the school pressure of college students. The stronger the job-searching self-efficacy, the weaker the impact of their perception reduction of employment opportunities on school pressure.

H3C: Job-searching self-efficacy negatively moderates the relationship between the perception reduction of employment opportunities under the COVID-19 epidemic and the family pressure of college students. The stronger the job-searching self-efficacy, the weaker the impact of their perception reduction of employment opportunities on family pressure.

#### The joint moderating effect of employment policy support and job-searching self-efficacy

According to the stress interaction theory, stress is a special relationship between individuals and the environment (Lazarus et al., 1985), and the employment stress of college students is affected by the interaction between the internal and external environment and personal factors (Liu, 2010). As an important environmental factor, employment policy support can alleviate employment pressure (OECD, 1993; Peng et al., 2020; Peng, 2021). As an important personal factor in the job search process of college students, a high level of job-searching self-efficacy can help individuals develop positive beliefs and enable individuals to have the ability and courage to face pressure (Yang et al., 2022). Resource Conservation Theory (COR) believes that the interaction between different resources and the impact on the environment is crucial. Hobfoll (2011) pointed out that different resources do not exist independently but are interconnected and impacted like a traveling “convoy,” and environmental factors such as the passage of the fleet also play an important role. Specifically, employment policy support and job-searching self-efficacy can be combined into four situations according to their level: (1) When the level of employment policy support and job-searching self-efficacy are both high, active employment policies provide college students with more information resources, job-searching opportunities and financial support can all reduce employment pressure (Peng, 2021), and college students with higher job-searching self-efficacy have stronger self-confidence and less job-searching anxiety (Wang and Cheng, 2022), in this case, even if employment opportunities are reduced under the epidemic, more external support and job-seeking confidence can ease the employment pressure of college students. (2) When the level of employment policy support and the level of job-searching self-efficacy are high and low, college students lack external environmental support or intrinsic motivational incentives. Although the employment pressure can be alleviated to a certain extent, the degree of relief will not reach the existing external support. And the level of inner self-confidence. (3) When the level of employment policy support and job-searching self-efficacy are both low, the employment policy is negative at this time, and college students can neither get support from the external environment in the process of job-searching, nor lack self-confidence in job-searching, so it is difficult to relieve the employment pressure.

H4: During the epidemic, the stronger the employment policy support and the higher the job-searching self-efficacy of college students, the weaker the impact of their perception reduction of employment opportunities under the COVID-19 epidemic on the employment pressure of college students.

H4A: During the epidemic, the stronger the employment policy support and the higher the job-searching self-efficacy of college students, the weaker the impact of their perception reduction of employment opportunities under the COVID-19 epidemic on the self-pressure of college students.

H4B: During the epidemic, the stronger the employment policy support and the higher the job-searching self-efficacy of college students, the weaker the impact of their perception reduction of employment opportunities under the COVID-19 epidemic on the school pressure of college students.

H4C: During the epidemic, the stronger the employment policy support and the higher the job-searching self-efficacy of college students, the weaker the impact of their perception reduction of employment opportunities under the COVID-19 epidemic on the family pressure of college students.

## Materials and methods

### Samples and research procedures

The subjects of the survey are mainly fresh graduates in 2020 and 2021. Because the final employment rate statistics of major universities are generally in August each year, the collection time of the questionnaires in this research is mainly concentrated in 2020 and 2021. This month, the questionnaires are mainly filled out by contacting fresh graduates of different universities through the social relations of the research team. By contacting the recruitment offices of major universities, counselors, teachers, and class teachers in charge of employment, electronic questionnaires are used, mainly in Sichuan, Chongqing, Shanghai, Jilin, Hunan, and other places to collect questionnaires. The distribution of the questionnaire is divided into three stages. First, the development of the new questionnaire and the test of reliability and validity. Because the two scales, the perception reduction of employment opportunities under the epidemic and the employment policy support under the epidemic, are both newly developed scales. Therefore, the main items of the two new scales were first determined by in-depth interviews and literature methods, and then 120 questionnaires were collected. Preliminary reliability and validity tests were performed on the two scales and related items were initially determined. Then, the research variables are pre-investigated. Before the questionnaire is distributed, the mature scale uses the translation-back translation program to translate the English items into Chinese and then asks several fresh college graduates to read the items and fully communicate, delete or revise them. For the ambiguous part, 213 questionnaires were collected for pre-investigation, and the questionnaire items and structural dimensions were revised again. Finally, a large-scale survey was conducted, and the study finally obtained 835 questionnaires. Invalid questionnaires were eliminated according to the completeness of the questionnaires, whether there were contradictions between positive and negative items, and whether the questionnaires were highly concentrated on one option. A total of 25 invalid questionnaires were eliminated. 810 valid questionnaires were obtained, and the effective rate of the sample was 97%. In the survey sample, females account for 40.4%, males account for 59.6%, rural students account for 69.6%, and urban residents account for 30.4%.

### Measuring tools

#### Dependent variable: Employment pressure of college students

The employment pressure scale developed by Liu (2010) has high reliability and validity and is widely used in China. The scale has 14 questions in four dimensions, i.e., family factors, school factors, professional factors, and self-factors. The following are some of the typical items for those four dimensions: the family’s economic foundation is not good; the school ranks relatively low; the employment rate of the major or corresponding major in the past is not good, and the person lacks experience of social practice. Measured by a five-point Likert scale, ranging from “1 (totally disagree)” to “5 (totally agree).”

#### Independent variable: Perception reduction of employment opportunities under the COVID-19 epidemic

Based on Zhao’s (2018) “Perception of Employment Opportunities,” through in-depth interviews with fresh college students and teachers in charge of employment, college students’ perception reduction of employment opportunities is measured from the reduction in the number and quality of employment during the epidemic. There are six questions in one dimension. There are six typical questions: the epidemic has reduced the number of my job interview; the epidemic has reduced the quality of my interviews; the epidemic has reduced the number of offers I get; the epidemic has reduced the quality of my offers; the epidemic has reduced the number of vacancies I can find, and the epidemic has reduced the quality of vacancies I can find. After testing, the scale has good reliability and validity. The Cronbach’s α of the scale is 0.948 (*n* = 810), the KMO value is 0.903 (*n* = 810), and the explained variance ratio is 79.634%, average extraction variance (AVE) = 0.7665, CR = 0.9514. Measured by a five-point Likert scale, ranging from “1 (totally disagree)” to “5 (totally agree).”

#### Moderating variables: Employment policy support and job-searching self-efficacy

##### Employment policy support under the COVID-19 epidemic

Based on Wang’s (2018) “employment policy support” scale, the background of the epidemic was added to adapt the questionnaire to measure the degree of support of the government and schools for college students’ employment under the epidemic. One dimension includes a total of three items. Typical questions include: “Many employment support policies issued by the state and local governments under the epidemic have helped me to some extent in employment,” “The current policy orientation under the epidemic provides strong support for employment and entrepreneurship,” and “Professional employment guidance and services provided by the university under the epidemic have provided support for my employment.” After testing, the scale has good reliability and validity. The Cronbach’s α coefficient of the scale is 0.803 (*n* = 810), the KMO value is 0.711 (*n* = 810), and the explained variance ratio is 71.84%, AVE = 0.549, CR = 0.7846. Measured by a five-point Likert scale, ranging from “1 (totally disagree)” to “5 (totally agree).”

#### Job-searching self-efficacy

The job-searching self-efficacy questionnaire compiled by Trougakos et al. (2007) has been verified to have good reliability and validity. The scale has 3 items and 1 dimension, with typical items such as “I know how to find a job that is currently hiring,” and “I know what type of job I want to apply for.” The Cronbach’s alpha of job search self-efficacy was 0.775 (*n* = 810), the KMO value was 0.699 (*n* = 810), and the explained variance was 70.453%; Measured by a five-point Likert scale, ranging from “1 (totally disagree)” to “5 (totally agree).”

#### Controlled variables

The controlled variables of this study mainly include gender, native place, human capital, and social capital. Due to occupational gender stereotypes and the physiological characteristics of women’s pregnancy and childbirth, gender is an important factor affecting employment pressure (Tang, 2021; Singh et al., 2022), so gender was used as a control variable, in which women were coded as 1 and men as 2. The place of origin is also an important factor affecting the employment pressure of college students (Hu et al., 2020), and the place of origin is coded as 1 for rural areas and 2 for urban areas. The measurement of human capital is combined with the research of Qiao et al. (2011) and Wang (2018), and the main indicators used in the measurement of human capital include 10 aspects: the level of graduates’ colleges, the level of English certificates obtained by college students, the level of computer certificates obtained by college students, students’ mastery of office software, students’ comprehensive evaluation score ranking, the number of professional qualification certificates, the highest level of scholarships obtained, internship experience, experiences in student associations, and the level of professional skills. The coding is coded from 1 to 5 according to the level from low to high, and then the average capital level of 10 human capital items is calculated by the continuous variable calculation method. Combined with Wang (2013, 2018) research, social capital is measured with five items and one dimension. Typical items are as follows: “I know a lot of people who are helpful to my job searching,” “most of the people who are helpful to my job searching have a good educational background and social status,” and “most of the people who are helpful to my job searching are people I know very well,” and measured by five-point Likert scale, ranging from “1 (totally disagree)” to “5 (totally agree).”

## Results

### Reliability analysis and common method variances test for the questionnaire

Taking into account the possible situational problems in the use of the translated questionnaire, about 50% of the large sample (*n* = 402) is firstly drawn for exploratory analysis. Cronbach’s α was used to test the reliability of the questionnaire, and the Cronbach’s α coefficients of several scales were obtained: the KMO value of the overall scale of employment stress is 0.800, the α value is 0.891, and the cumulative explained variance ratio is 69.326%; the KMO value of family stress is 0.615, the α value is 0.674, and the cumulative explained variance ratio is 51.662%; the KMO value of school pressure is 0.754, the α value is 0.839, and the cumulative explained variance ratio is 68.229%; the KMO value of professional pressure is 0.748, the α value is 0.865, and the cumulative explained variance ratio is 78.820%; the KMO value of self-pressure is 0.670, the α value is 0.839, and the cumulative explained variance ratio is 75.777%; the KMO value of perception reduction of employment opportunities is 0.896,the α value is 0.952, and the explained variance is 80.796%; the job-searching self-efficacy KMO value is 0.697, the α value is 0.770, and the explained variance is 70.453%; the KMO value supported by the employment policy under the epidemic is 0.702, the α value is 0.789, and the cumulative explained variance ratio is 65.429%; the KMO value of social capital is 0.652, the α value is 0.675, and the cumulative explained variance ratio is 65.429%. From the above description, we can see that the Cronbach’s α value of all variables is higher than 0.6, and the KMO value is higher than 0.6, indicating that the questionnaire has good reliability and is suitable for factor analysis. Since the controlled variable, human capital, is measured by specifically related indicators, there is no need for reliability and validity testing.

Since most questionnaires use self-rating scales, to control the variance of the same origin, the participants are told that this would be an anonymous survey and that the questionnaire would only be used for scientific research purposes, to dispel the participants’ doubts and reflect on their true situation. At the same time, the Harman single factor test method is used to test the homology variance, and all the items in the questionnaire are analyzed by principal component analysis. It is found that the first principal component explains 22.693% of the variance, which is far less than the recommended value of 50%.

### Confirmatory factor analysis

The exploratory factor analysis shows that the structural dimensions of each variable are consistent with the previous theoretical assumptions. Then AMOS6.0 is adopted to perform confirmatory factor analysis on about 50% of the other half of the samples (*n* = 408). The specific data are shown in Table 1.

**TABLE 1:** Results of confirmatory factor analysis of self-compiled scale (*n* = 408).

Latent variable	Item	Standard load	AVE	Suggested value of AVE	CR	Suggested value of CR
Perception reduction of employment opportunities under the COVID-19 epidemic	The epidemic has reduced my number of job interviews.	0.75	0.7665	≥0.5	0.9514	≥0.6
	The epidemic has reduced the number of offers I get.	0.85				
	The epidemic has reduced the number of vacancies I can find.	0.88				
	The epidemic has reduced the quality of my interview companies.	0.91				
	The epidemic has reduced the quality of my offers.	0.92				
	The epidemic has reduced the quality of vacancies I can find.	0.93				
	During the epidemic, the national and local governments have introduced many employment support policies that have helped my employment.	0.78	0.549		0.7846	
Employment policy support	Current policy guidance under the epidemic strongly supports employment and entrepreneurship.	0.75				
	Under the epidemic, the school’s specialized employment guidance and services provided support for my employment.	0.69				

Since the remaining scales are all mature scales, and the two scales, “the perception reduction of employment opportunities under the COVID-19 epidemic” and “employment policy support under the COVID-19 epidemic,” are both self-developed scales, to further verify their validity, AMOS is used to carry out a confirmatory factor analysis on them, as shown in Table 1. Among them, the AVE of the perception reduction of employment opportunities scale is 0.7665, and the AVE of the employment policy support is 0.549, both of which are greater than 0.5 (Fornell and Larcker, 1981), which meets the requirements. The combined reliability (CR) of the perception reduction of employment opportunities scale is 0.9514, and the combined reliability of employment policy support under the epidemic is 0.7846, both of which are greater than 0.6 (Fornell and Larcker, 1981), which meets the requirements. This shows that the two self-compiled scales have good internal consistency.

In addition to the basic model containing five variables, the study also assumes five alternative models to compare the advantages and disadvantages of the models. The specific data are shown in Table 2. All key indicators in the basic model are higher than 0.85. Except for AGFI, the rest of the fitting indexes in the basic model are all above 0.9 and are better than other models’ fitting indexes. Therefore, it shows that the scale has good discriminative validity.

**TABLE 2:** Confirmatory factor analysis results (*n* = 408).

Model	Description	χ^2^/df	CFI	NFI	IFI	GFI	AGFI	RMSEA
Basic model	Six-factor model	2.052	0.958	0.921	0.958	0.912	0.886	0.051
Model 1	Five-factor model	3.792	0.885	0.852	0.886	0.831	0.784	0.083
Model 2	Four-factor model	5.010	0.833	0.801	0.834	0.767	0.708	0.099
Model 3	Three-factor model	5.966	0.791	0.760	0.792	0.738	0.674	0.110
Model 4	Two-factor model	8.372	0.687	0.661	0.689	0.661	0.582	0.135
Model 5	Single-factor model	10.224	0.607	0.584	0.609	0.613	0.525	0.151

The basic model is the hypothetical model for this research (excluding controlled variables). Model 1 is the perception reduction of employment opportunities + employment policy support, job-searching self-efficacy, family pressure, school pressure, and self-pressure. Model 2 is the perception reduction of employment opportunities + employment policy support + job-searching self-efficacy, family pressure, school pressure, and self-pressure. Model 3 is the perception reduction of employment opportunities + employment policy support + job-searching self-efficacy + family pressure, school pressure, and self-pressure. Model 4 is the perception reduction of employment opportunities + employment policy support + job self-efficacy + family pressure + school pressure, and self-pressure. Model 5 is the perception reduction of employment opportunities + employment policy support + job self-efficacy + family pressure + school pressure + self-pressure.

### Descriptive statistical analysis

Table 3 shows the correlation coefficient, and the mean and standard deviation of the main research variables. Among them, the first row’s 1–10, respectively indicate gender, native place, human capital, social capital, the perception reduction of employment opportunities, employment policy support under the epidemic, job-searching self-efficacy, family pressure, and school pressure, and self-pressure.

**TABLE 3:** Means, standard deviations, and correlation analysis results of various variables (*n* = 810).

Variable	1	2	3	4	5	6	7	8	9	10
Gender	1									
Native Place	−0.004	1								
Human Capital	0.247**	0.035	1							
Social Capital	−0.088*	0.069*	0.064	(0.657)						
Perception reduction of employment opportunities	0.236**	−0.032	0.113**	−0.036	(0.948)					
Employment policy support	−0.050	−0.043	0.050	0.411**	−0.137**	(0.803)				
Job-searching self-efficacy	−0.057	0.084*	0.042	0.317**	−0.040	0.343**	(0.770)			
Family pressure	0.025	−0.222**	0.008	0.007	0.182**	0.038	0.015	(0.699)		
School pressure	0.041	−0.003	−0.005	−0.280**	0.346**	−0.294**	−0.138**	0.077*	(0.880)	
Self-pressure	0.135**	−0.095**	−0.101**	−0.118**	0.232**	−0.017	−0.305**	0.163**	0.302**	(0.836)
Mean	1.6	1.3	2.648	3.2886	3.2325	3.284	3.2864	3.4539	2.6453	3.0728
Standard deviation	0.491	0.46	0.55083	0.6113	0.99289	0.72207	0.67648	0.81725	0.77726	0.85594

***p < 0.001, **p < 0.01, *p < 0.05. The data on the diagonal is the Cronbach’s Alpha value of the scale. Among them, the first row’s 1–10, respectively indicate gender, native place, human capital, social capital, perception reduction of employment opportunities under the COVID-19 epidemic, employment policy support, job-searching self-efficacy, family pressure, school pressure, and self-pressure.

There is a significant difference in the perception reduction of employment opportunities (β = 0.236; *p* < 0.01) and self-pressure (β = 0.135; *p* < 0.01) between different genders. Males perceive fewer employment opportunities under the epidemic and greater pressure on themselves, and this may be related to the higher social status and requirements of men.

There is a significant difference in job-searching self-efficacy between different native places (β = 0.084; *p* < 0.05). College students with urban registered residence have a stronger sense of self-efficacy in job searching and show more confidence in job hunting. Similarly, native places show significant differences in the performance of family stress and self-stress (family pressure: β = −0.222; *p* < 0.01; self-pressure: β = −0.095; *p* < 0.01). It is manifested that the family pressure and self-pressure of college students with rural registered residence are greater than those with urban registered residence, particularly family pressure the strongest.

Human capital is positively correlated with the perception reduction of employment opportunities (β = 0.113; *p* < 0.01), which shows that college students with better academic performance, more certificates, awards, and internships perceive a greater impact of the epidemic on employment. This may be related to the higher expectations of these college students for their employment; at the same time, human capital is negatively correlated with self-pressure (β = −0.101; *p* < 0.01), which also shows that college students with good learning, more certificates, awards, and internships do not panic when they are well prepared, and they will have less self-pressure and employment pressure.

However, social capital is positively correlated with the sense of employment policy support (β = 0.411; *p* < 0.01) and job-searching self-efficacy (β = 0.317; *p* < 0.01), indicating that college students with more social relationship resources perceived a higher degree of policy support, higher job-searching self-efficacy and more confidence in job searching. At the same time, the employment pressure from their school and family is lower (β = −0.280; *p* < 0.01), and the pressure from oneself is also lower (β = −0.118; *p* < 0.01).

At the same time, according to Table 3, it can be seen that the perception reduction of employment opportunities is significantly positively correlated with students’ self-pressure (β = 0.232; *p* < 0.01), school pressure (β = 0.346; *p* < 0.01), and family pressure (β = 0.182; *p* < 0.01), which preliminarily shows that the higher the perception reduction of employment opportunities perceived by college students, the greater their employment pressure.

### Hypothesis testing

The study uses a multi-level regression method to test the main effects of the perception reduction of employment opportunities on college students’ family pressure, school pressure, and self-pressure, and to verify the independent moderating effects of employment policy support and job-searching self-efficacy, respectively on the three main effects. Finally, verify the joint adjustment effect of the two moderating variables on the relationship between the perception reduction of employment opportunities and students’ pressure from their family, school, and themselves. According to the suggestions of Aiken et al. (1991), Cohen et al. (2003), and Hayes (2013), to make the coefficients of the regression equation more explanatory, all variables should be centralized before the analysis. Under the epidemic, the perception reduction of employment opportunity is represented by REO, employment policy support is represented by EPS, and job-searching self-efficacy is represented by JS.

The Dawson (2014) method was used to test individual and joint moderating effects. When examining the independent moderating effects of employment policy support and job-seeking self-efficacy under the epidemic, Eq. 1 is used, where y represents the dependent variable (employment pressure), x represents the independent variable (perception reduction of employment opportunities under the COVID-19 epidemic), and z represents the adjustment Variables (employment policy support under the COVID-19 epidemic or job-searching self-efficacy under the epidemic).


(1) $$\text{y}={\text{b}}_{0}+{\text{b}}_{1}\,\text{x}+{\text{b}}_{2}\,z+{\text{b}}_{3}\,\text{xz}+\varepsilon$$

When examining the joint moderating effect of employment policy support and job-seeking self-efficacy under the epidemic, Eq. 2 is used, where y represents the dependent variable (employment pressure), x represents the independent variable (perception reduction of employment opportunities under the COVID-19 epidemic), and z and w represent Two moderating variables (employment policy support under the COVID-19 epidemic or job-searching self-efficacy under the epidemic).


(2) $$\text{y}={\text{b}}_{0}+{\text{b}}_{1}\,\text{x}+{\text{b}}_{2}\,\text{z}+{\text{b}}_{3}\,\text{w}+{\text{b}}_{4}\,\text{xz}+{\text{b}}_{5}\,\text{xw}+{\text{b}}_{6}\text{zw}+{\text{b}}_{7}\,\text{xzw}+\varepsilon$$

### Independent and joint moderation of employment policy support and job-searching self-efficacy on students’ perception reduction of employment opportunities under the COVID-19 epidemic and self-pressure

As shown in Models 1 and 2 in Table 4, when only the controlled variables are considered, the perception reduction of employment opportunities has a significant positive impact on the self-pressure of college students (β = 0.186; *p* < 0.01), and hypothesis 1A is supported. It can be seen from Models 1, 2, 3, and 4 that the product term of perception reduction of employment opportunities and employment policy support is not significant (β = 0.002; *p* > 0.1), so hypothesis 2A is not supported. Models 1, 2, 5, and 6 show that the product term of perception reduction of employment opportunities and job-searching self-efficacy is significant (β = 0.046; *p* < 0.01), and job-searching self-efficacy support negatively moderates the relationship between perception reduction of employment opportunities and self-pressure. The stronger the job-searching self-efficacy among college students, the weaker the impact of their perception reduction of employment opportunities on self-pressure. Therefore, hypothesis 3A is verified. From Models 7, 8, and 9, it is seen that the triple interaction coefficient of perception reduction of employment opportunities, employment policy support, and job-searching self-efficacy is significant (β = 0.034; *p* < 0.05), and the triple interaction adjustment effect exists, that is, the stronger the employment policy support, the higher the college students’ job-searching self-efficacy, and the greater the impact of the perception reduction of employment opportunities on the self-pressure of college students. Hypothesis 4A has been verified.

**TABLE 4:** The moderating effect of employment policy support and job-searching self-efficacy on the relationship between perception reduction of employment opportunities and self-pressure.

Dependent variable: Self-pressure									
Variable	Model 1	Model 2	Model 3	Model 4	Model 5	Model 6	Model 7	Model 8	Model 9
Constant coefficient	2.832	2.345	2.307	2.307	2.346	2.354	2.264	2.276	2.304
Gender	0.278***	0.195**	0.194**	0.194**	0.179**	0.175**	0.176**	0.168**	0.182**
Native place	−0.155**	−0.143**	−0.132**	−0.132**	−0.111*	−0.109*	−0.085	−0.085	−0.082
Human capital	−0.113***	−0.125***	−0.127***	−0.127**	−0.117***	−0.117***	−0.121***	−0.122***	−0.125***
Social capital	−0.077**	−0.074**	−0.099**	−0.099***	0.000	0.000	−0.044	−0.043	−0.045
REO		0.186***	0.194***	0.195	0.181***	0.181***	0.198***	0.200***	0.181***
EPS			0.061*	0.055			0.132***	0.090	0.093
JS					−0.240***	−0.384***	−0.271***	−0.373***	−0.394***
REO × EPS				0.002				0.013	0.012
REO × JS						0.046**		0.031	0.041*
EPS × JS								−0.015	−0.109**
REO × EPS × JS									0.034**
*R* ^2^	0.053	0.097	0.101	0.101	0.167	0.172	0.186	0.189	0.197
*R*^2^ changes		0.044	0.004	0.000	0.070	0.004	0.132	0.004	0.008
*F*	11.364***	17.353***	15.114***	12.940***	26.922***	23.785***	26.097***	18.670***	17.826***
*F* changes		39.156***	3.638**	0.007	67.582***	4.301*	43.351***	1.276	7.798**

***p < 0.001, **p < 0.01, *p < 0.05. All the coefficients in the table are non-standardized coefficients after data centralization. In the subsequent interaction effect diagram, the dependent variable adopts non-centralized data to better observe the constant term (the same below). Among them, the change of R^2^ and the change of F, respectively represent the change from Model 1 to Model 4, which tests the moderating effect of employment policy support. The changes from models 1, 2 to model 5, 6 tested the moderating effect of job-searching self-efficacy; the changes from model 7 to model 9 tested three interactive moderating effects.

**FIGURE 2:**
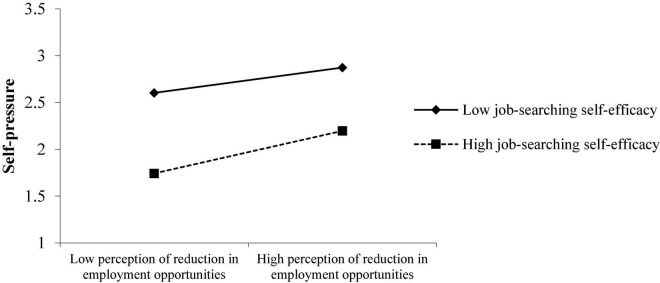
Role of job-searching self-efficacy in moderating the perception reduction of employment opportunities and college students’ self-pressure.

In this paper, the adjusted variable is divided into groups of high scores and groups of low scores by adding and subtracting one standard deviation from the average of the adjusted variable, and then graphs are drawn. It can be seen from Figure 2 that job-searching self-efficacy negatively moderates the impact of the perception reduction of employment opportunities on college students’ employment pressure. When college students with higher job-searching self-efficacy face reduced job opportunities under the epidemic situation, their pressure is significantly lesser than that of college students with lower job-searching self-efficacy.

**FIGURE 3:**
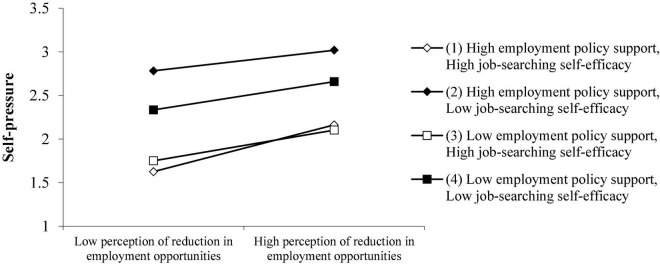
The joint-moderating effect of employment policy support and job-searching self-efficacy on the perception reduction of employment opportunities and employment pressure of college students.

It can be seen from Figure 3 that the higher the degree of employment policy support under the epidemic and the higher the job-searching self-efficacy, the lower the self-pressure of college students. Especially when the job-searching self-efficacy is high, this performance is more obvious. At the same time, it can be seen from Figure 3 that when the perception reduction of employment opportunities brings the greatest employment pressure to college students, it is not when the two variables are low, but when the employment policy support is high and the job-searching self-efficacy is low. This phenomenon may be caused by the fact that college students perceive that the external employment support policy is very strong, but they are not confident enough in job hunting so the pressure of external expectations and the pressure of not having enough self-confidence in job hunting are superimposed on each other, which will lead to greater self-pressure of college students.

### Independent and joint moderation of employment policy support and job-searching self-efficacy on students’ perception reduction of employment opportunities under the COVID-19 epidemic and school pressure

As shown in Model 11 in Table 5, when only the controlled variables are considered, the perception reduction of employment opportunities has a significant positive effect on school pressure (β = 0.276; *p* < 0.01). Hypothesis 1B is supported. It can be seen from Models 11, 12, and 13 that the product term of perception reduction of employment opportunities and employment policy support is not significant (β = −0.010; *p* > 0.1), so hypothesis 2B is not supported. It can be seen from Models 10, 11, 14, and 15 that the product term of perception reduction of employment opportunities and job-searching self-efficacy is not significant (β = 0.012; *p* > 0.1). Therefore, hypothesis 3B has not been verified. Models 16, 17, and 18 show that the perception reduction of employment opportunities, employment policy support and job-searching self-efficacy’s triple interaction coefficient is significant (β = 0.027; *p* < 0.01), and the triple interaction adjustment effect exists, that is, the stronger the employment policy support under the epidemic, the higher students’ job-searching self-efficacy, and the weaker the impact of the perception reduction of employment opportunities on the school pressure. Therefore, Hypothesis 4B is verified.

**TABLE 5:** The moderating effect of employment policy support and job-searching self-efficacy on the perception reduction of employment opportunities and school pressure.

Dependent variable: School pressure									
Variable	Model 10	Model 11	Model 12	Model 13	Model 14	Model 15	Model 16	Model 17	Model 18
Constant coefficient	2.573	1.850	1.930	1.928	1.850	1.852	1.929	1.939	1.962
Gender	0.023	−0.100*	−0.098*	−0.098*	−0.102*	−0.103*	−0.098*	−0.104*	−0.093*
Native place	0.028	0.047	0.025	0.026	0.052	0.052	0.027	0.026	0.028
Human capital	0.007	−0.010	−0.005	−0.005	−0.009	−0.008	−0.005	−0.005	−0.007
Social capital	−0.218***	−0.213***	−0.160***	−0.160***	−0.201***	−0.201***	−0.159***	−0.158***	−0.159***
REO		0.276***	0.259***	0.260***	0.276***	0.276***	0.260***	0.261***	0.246***
EPS			−0.129***	−0.159**			−0.127***	−0.142**	−0.140**
JS					−0.037	−0.076	−0.007	−0.065	−0.082
REO × EPS				0.010				0.005	0.004
REO × JS						0.012		0.018	0.025
EPS × JS								−0.014	−0.090**
REO × EPS × JS									0.027**
*R* ^2^	0.079	0.196	0.218	0.218	0.198	0.198	0.218	0.220	0.226
*R*^2^ changes		0.117	0.022	0.000	0.002	0.000	0.139	0.002	0.006
*F*	17.220***	39.2***	37.358***	32.032***	33.052***	28.364***	47.698***	22.549***	21.223***
*F* changes		117.182***	22.828***	0.273	2.052	0.389	319.994***	0.617	6.429**

***p < 0.001, **p < 0.01, *p < 0.05. Among them, the change of R^2^ and the change of F, respectively represent the change from model 10 to model 13, which tests the moderating effect of employment policy support. The changes from models 10, 11 to models 14, 15 tested the moderating effect of job-searching self-efficacy; the changes from model 16 to model 18 tested three interactive moderating effects.

**FIGURE 4:**
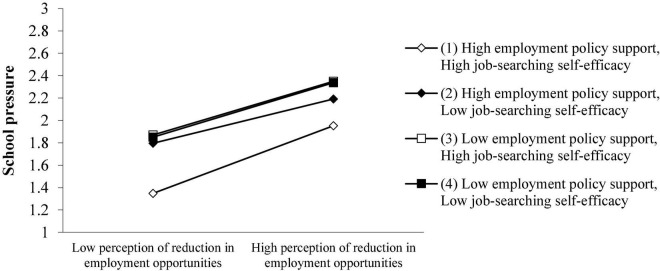
Employment policy support and job-searching self-efficacy have a joint-moderating effect on the perception reduction of employment opportunities and college students’ school pressure.

It can be seen from Figure 4 that when college students perceive that employment policy support and job-searching self-efficacy are both high, the perception reduction of employment opportunities will have the least employment pressure brought by the school; also, policy support and job-searching self-efficacy can effectively alleviate the employment pressure caused by colleges or universities.

### Independent and joint moderation of employment policy support and job-searching self-efficacy on students’ perception reduction of employment opportunities under the COVID-19 epidemic and family pressure

As shown in Model 20 in Table 6, when only the controlled variables are considered, the perception reduction of employment opportunities has a positive and significant impact on the pressure of college students from their families (β = 0.148; *p* < 0.01), and hypothesis 1C is supported. From models 20, 21, and 22, it can be seen that the product term of perception reduction of employment opportunities and employment policy support is significant (β = −0.055; *p* < 0.05). It can be seen from models 19, 20, 23, and 24 that the product term of perception reduction of employment opportunities and job-searching self-efficacy is significant (β = −0.047; *p* < 0.05). From models 25, 26, and 27, it can be seen that the triple interaction coefficients of perception reduction of employment opportunities, employment policy support, and job-searching self-efficacy under the epidemic are significant (β = 0.057; *p* < 0.01), and the triple interaction adjustment effect exists.

**TABLE 6:** The moderating effect of employment policy support and job-searching self-efficacy on the perception reduction of employment opportunities and family pressure during the epidemic.

Dependent variable: Family pressure									
Variable	Model 19	Model 20	Model 21	Model 22	Model 23	Model 24	Model 25	Model 26	Model 27
Constant coefficient	3.909	3.520	3.494	3.505	3.520	3.512	3.498	3.519	3.567
Gender	0.041	−0.025	−0.026	−0.024	−0.023	−0.019	−0.024	−0.031	−0.007
Native place	−0.398***	−0.388***	−0.381***	−0.384***	−0.392***	−0.394***	−0.385***	−0.392***	−0.387***
Human capital	0.006	−0.003	−0.004	−0.001	−0.004	−0.004	−0.005	−0.003	−0.008
Social capital	0.020	0.023	0.006	0.005	0.014	0.014	0.002	−0.004	−0.007
REO		0.148***	0.154***	0.149***	0.149***	0.149***	0.154***	0.155***	0.123***
EPS			0.042	0.211***			0.036	0.188**	0.192**
JS					0.028	0.176**	0.019	0.146*	0.109
REO × EPS				−0.055**				−0.046**	−0.048**
REO × JS						−0.047**		−0.040*	−0.024
EPS × JS								−0.045**	−0.205***
REO × EPS × JS									0.057***
*R* ^2^	0.051	0.081	0.083	0.091	0.082	0.087	0.084	0.089	0.125
*R*^2^ Changes		0.031	0.002	0.007	0.001	0.005	0.033	0.017	0.025
*F*	10.754***	14.228***	12.175***	11.440***	12.009***	10.984***	10.487***	8.930***	22.545***
*F* Changes		26.747***	1.835	6.528**	0.925	4.516**	9.668***	4.936**	10.386***

***p < 0.001, **p < 0.01, *p < 0.05. Among them, the change of R^2^ and the change of F, respectively represent the change from model 19 to model 22, which tests the moderating effect of employment policy support. The changes from models 19, 20 to models 23, 24 tested the moderating effect of job-searching self-efficacy; the changes from model 25 to model 27 tested the three interactive moderating effects.

**FIGURE 5:**
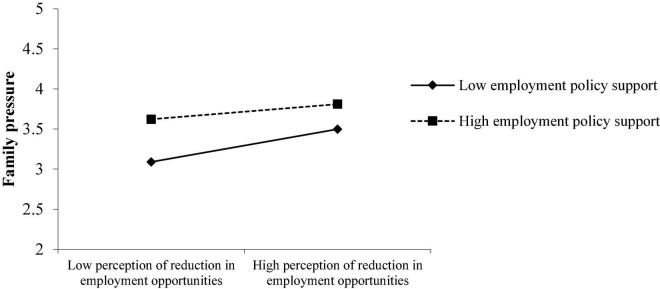
The role of employment policy support in moderating the perception reduction of employment opportunities on the employment pressure of college students’ families.

As shown in Figure 5, what is inconsistent with the hypothesis is that when the employment policy support increases during the epidemic, the employment pressure of college students from their families has not weakened but increased. According to the Expectation Theory, when the employment support policy becomes stronger under the epidemic situation, college students themselves, including college students’ families, will feel that since so much policy support has been implemented, students should be able to get better employment, which objectively increases graduates’ employment pressure from their families. The more the policy support provided by the external environment, the greater the possible employment pressure from students’ families. At the same time, combined with the observation of the average value of each variable in Table 3, it can be found that the employment pressure from the family is the largest among all types of employment pressures, with an average value of 3.4539. In contrast, the average employment pressure from school is 2.6453. The average employment pressure from students themselves is 3.0728. China has a traditional saying of “expecting the boy to become a dragon and the girl to become a phoenix,” meaning “wishing one’s children a promising future.” What college students cannot bear is mainly the pressure from their parents’ hard work to raise them and hopes for their future. Once the expectations of their parents or family are not fulfilled, the pressure on college students will be even greater.

**FIGURE 6:**
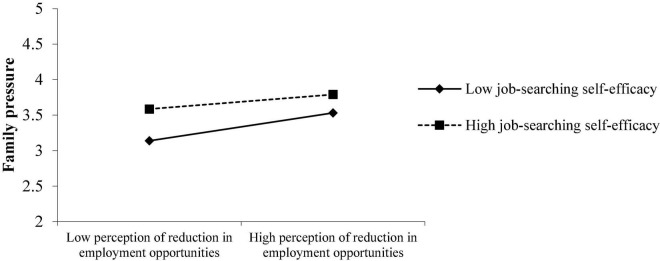
The role of job-searching self-efficacy on the perception reduction of employment opportunities on the employment pressure of college students’ families.

As shown in Figure 6, what is inconsistent with the hypothesis is that when job-searching self-efficacy increases, the employment pressure of college students from their families has not weakened but increased. As shown in Figure 5, when college students themselves are more confident in job hunting, since they and their families have higher expectations, they will objectively feel more pressure from their families when they are employed. If they do not find a good job, they will feel sorry for their parents’ hard-working to support them. Therefore, college students who have been more confident in their job searching may have greater employment pressure from their families.

**FIGURE 7:**
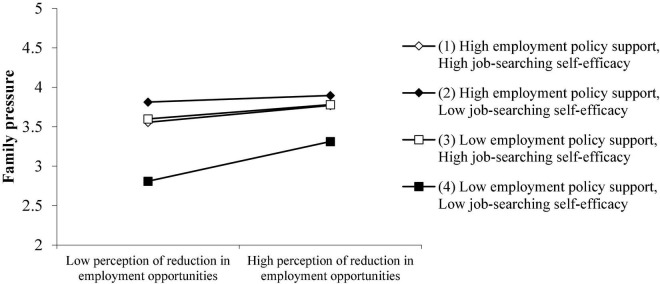
The joint-moderating effect of employment policy support and job-searching self-efficacy on the perception reduction of employment opportunities and family pressure.

As shown in Figure 7, what is inconsistent with the assumption is that when the employment policy support is weaker under the epidemic situation, and the job-searching self-efficacy is weaker, the perception reduction of employment opportunities has the least impact on the employment pressure of the family. According to the Expectation Theory, when employment policy support is strong and college students themselves are more confident in job hunting since they and their families have higher expectations, they will objectively feel more pressure from their families when they are employed. If they neither perceive much support from external policies in the employment process nor have low self-confidence in the employment process, their families are not likely to have requirements and expectations that are too high for them. Thus college students may have less employment pressure from their families.

## Discussion

### Research conclusion

The research is based on the stress interaction theory, by constructing a theoretical model of the relationship between the perception reduction of employment opportunities and college students’ employment pressure under the epidemic and using 810 fresh college students’ survey data for empirical analysis, and the following conclusions are obtained: First, the perception reduction of employment opportunities under the COVID-19 epidemic is significantly positively correlated with college students’ Self-pressure, school pressure, and family pressure. Compared with their own employment pressure and school employment pressure, college students’ employment pressure from their families is greater. Second, under the epidemic, employment policy support and job-searching self-efficacy negatively moderate the relationship between the perception reduction of employment opportunities and one’s own employment pressure and school employment pressure. When employment policy support and job-searching self-efficacy are both high under the epidemic, there will be less pressure from college students themselves and their schools. Third, employment policy support and job-searching self-efficacy under the epidemic positively moderate the relationship between the perception reduction of employment opportunities and family employment pressure. When employment policy support and job-searching self-efficacy are both high during the epidemic, college students perceive the least employment pressure from their families. Fourth, the employment policy support and the coexistence of internal and external causes of college students’ professional self-efficacy under the epidemic can alleviate the employment pressure from college students themselves and the school. However, when college students themselves and their families have given the external policy support expectations that are too high, it will intensify the employment pressure of the college students from their families.

### Theoretical significance

First, it enriches the content of psychological research from the perspective of college students’ employment pressure. Although previous studies have paid attention to the changes in employment pressure and psychological pressure of college students under the epidemic (Li, 2020), as the most important source of stress affecting college students’ mental health (Liu, 2019), they have not deeply explored the mechanism of the perceived reduction of employment opportunities on college students’ employment stress under the epidemic. Combined with the special background of the epidemic, the research analyses the impact of perception reduction of employment opportunities on three different types of college students’ employment pressure and conducts an empirical test, which enriches the research on the impact of the epidemic on college students’ employment pressure and expands the content of psychological research.

Secondly, the boundary conditions of employment pressure research are expanded from the perspective of the external environment and personal characteristics. The previous research on the employment pressure of college students mainly focused on the influencing factors of the employment pressure of college students (Tang and Sun, 2021), and there was almost no research on the boundary conditions of the employment pressure of college students. This study focuses on the important external environment of employment policy support and the important personal characteristics of college students’ job-searching self-efficacy, discusses the boundary mechanism of the impact of perception reduction of employment opportunities on college students’ employment pressure under the epidemic, and in-depth analyses the independent and joint moderating effects, expands the research on contingency factors of employment pressure.

Finally, we deeply analyze the different effects of employment policy support and job-searching self-efficacy on different relationships. The study found that employment policy support and job-searching self-efficacy negatively moderated the relationship between perception reduction of employment opportunities and their own employment pressure and school employment pressure during the epidemic, but positively moderated the relationship between perception reduction of employment opportunities and family pressure, revealing the complex mechanism of perception reduction of employment opportunities on different employment pressures.

### Practical inspiration

First of all, the government, families, and schools should pay attention to the employment pressure and mental health of college students under the epidemic. Employment is the primary way for college students to enter society to realize their own value, and employment pressure is the most important source of influence on college students’ mental health (Liu, 2019). However, under the epidemic, employment opportunities have decreased, and college students are facing greater employment pressure. Families and society must not place high expectations on the employment of college students, especially in the Chinese culture that emphasizes “filial piety.” Most college students think that it is not easy for their parents to support them in college, so they especially hope to find a good job to repay their parents and families, which undoubtedly increases. Invisible pressure from family when graduates are employed.

Second, the government and universities should introduce active employment support policies. Stock (2020) pointed out that although different policy responses can achieve similar public goals, their effects can vary widely. Judging from the aforementioned research, employment policy support can significantly alleviate various employment pressures for college students. Although it may increase the pressure of college students from their families due to excessive expectations, it still provides necessary environmental support and policy guarantees for helping college students to find employment;

Finally, the confidence level of college students in the job search process is crucial. Job-searching self-efficacy can significantly relieve college students of various employment pressures. It is very important to understand and know how to find a matching position, to know the type of job they want to apply for, to clarify their employment goals, and to enhance their self-confidence in employment. Turning employment pressure into a driving force for employment, making full use of various employment policies and external resources, and enhancing their own employment skills, can college students find their own foothold in the competitive employment market.

### Research limitations and prospects

First, the stability and reliability of the new scale need to be further examined. Since there are few studies on the employment of college students during the epidemic, and there are no mature scales for some variables, this study developed two new scales, the perception reduction of employment opportunities under the COVID-19 epidemic and the employment policy support under the epidemic. These two scales all passed the reliability and validity test, but more research is needed to test the stability and reliability of the reliability and validity of the questionnaire.

Second, the study sample and data evaluation methods have limitations. The research has only collected 810 survey data through team resources. In the future, it should cooperate with large employment research institutions to obtain big data, or use scientific sampling methods to expand the sampling scope, so that the research has better external validity. In addition, the research data were obtained through the self-assessment of college students, which may have endogeneity problems to a certain extent, but the results of Harman single factor analysis and confirmatory factor analysis confirmed that there was no serious common method bias. In future research, according to the nature of the variable itself, data can be collected through more different methods and sources. For example, data of different variables can be collected in stages at different time nodes. For example, employment policy support can be evaluated by objective data or from households, Multi-party evaluation data for schools.

Finally, the study explores the boundary mechanism in depth but does not analyze the mediation mechanism. The study explores the contingency effects of two important variables of college students’ job-searching self-efficacy and employment policy support but does not delve into why the perceived reduction of employment opportunities will further aggravate college students’ employment pressure, this “black box” needs to be further explored in the future.

## Data availability statement

The raw data supporting the conclusions of this article will be made available by the authors, without undue reservation.

## Author contributions

SY and LY: writing, model, and data. JY: model. JX: revise the manuscript. XL: translate this manuscript. WL, HC, and GH: collecting data. All authors contributed to the article and approved the submitted version.
